# Detection of selected antimicrobial residues in red meat and kidney of beef cattle slaughtered at Nekemte municipal abattoir, Ethiopia

**DOI:** 10.1002/vms3.1459

**Published:** 2024-04-24

**Authors:** Debela Abdeta, Mathewos Tafesse, Balachew Bacha

**Affiliations:** ^1^ College of Veterinary Medicine and Agriculture Addis Ababa University, Bishoftu Oromia Region Ethiopia; ^2^ Guto Gida district Livestock Health expert, Nekemte Oromia Region Ethiopia; ^3^ Ethiopian Agricultural Authority Animal Products and Inputs Quality Testing center(EAA‐APIC) Addis Ababa Ethiopia

**Keywords:** antibiotic residue, cattle, oxytetracycline, beef

## Abstract

**Background:**

Antibiotics are commonly used drugs in farm animals for therapeutic, prophylactic and diagnosis purposes. As a result of the use of antibiotics in livestock, residues of antibiotics may be present in animal‐derived foods, especially in meat. This study aimed at determining the antibiotic residues in cattle slaughtered at Nekemte municipal abattoir and assessing the knowledge, attitude and practice (KAP) level of the community about antibiotic residue in food of cattle origin.

**Materials and methods:**

A cross‐sectional study was conducted on randomly selected kidney and muscle samples slaughtered at Nekemte municipal abattoir. The samples were collected aseptically and analysed using liquid chromatography mass spectrometer. In addition the KAP of cattle handlers, butcher men and meat users were collected using structured questionnaire. The data was analysed by SPSS and intercooled Stata version 7.0, 2001.

**Results:**

Results are presented as percentages and frequency distributions in tabular and graphical form. From 120 individuals interviewed, only 25.83% knew about withdrawal time and had limited knowledge on effect of antibiotic residue on human health, whereas about 47.5% did not heard about antibiotic residue in meat. Tetracycline was detected in all the sampled meat 60 (100%), whereas oxytetracycline residues were detected in half 15 (50%) of the samples. In the current study doxycycline, sulphadiazine, penicillin G and enrofloxacin were not detected in all samples. Oxytetracycline residue levels ranged from 0.00 to 463.35 µg/kg for the kidney and 0.00 to 354.55 µg/kg for muscle samples. About 10% of kidney and 3.33% muscle samples collected had oxytetracycline residues above maximum residue limits.

**Conclusion:**

In general, the study revealed that oxytetracycline residues were prevalent among antimicrobial residues analysed from the study area. The study indicated the presence of high antimicrobial residue and hence exposes for antimicrobial resistance of pathogens warranting coordinated effort to mitigate its health effect on the animal and hence human being.

## Graphical abstract

Antibiotic residue builds up as a result of the continuous use of antibiotics like oxytetracycline or tetracycline to cure illnesses, prevent the spread of diseases, and promote animal growth.

Residual accumulation in animal source food will lead to different adverse effects to humans and animals.

The low dose exposure by microorganisms leads development of antimicrobial resistance.

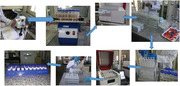

## 1 INTRODUCTION

Food safety is of great importance to consumers’ health. Currently, hundreds of substances are used as growth enhancers, prophylaxis and management of diseases in food‐producing animals (Shahbazi et al., 2015). Antibiotics are among the most widely used drugs. Previous studies have confirmed the inappropriate use of these drugs in animals by livestock owners and pastoralists (Alhaji & Ishola, 2018). The overuse of antibiotics in animals has been connected to the build‐up of antibiotic residues in foods derived from animals used for human consumption as well as to the selection pressure for bacteria resistant to antibiotics in both people and animals. The overuse of antibiotics in animals leaves residues in their meat, milk and eggs that eventually find their way into human bodies through bioaccumulation. These residues can be carcinogenic, mutagenic, teratogenic, nephrotoxic, hepatotoxic, bone marrow toxicity or allergic reaction (Nisha, 2008).

Even if there is little data on the true number of people or animals that use drugs irrationally, government authorities’ responses to the issue do not seem credible. Study conducted by Melku et al. (2021) in Ethiopia indicates that about half of the drugs are used indiscriminately. Furthermore, there is a lack of awareness and preparedness in dealing with the risk of indiscriminate use of antimicrobials. Food animals slaughtered for domestic and export purposes in the country are not screened for the presence of residues in any of the slaughterhouses. Few studies conducted in Ethiopia provide the information on the level of antibiotics residues (Bedada & Zewde, 2012) where misuse of antibiotics is practiced.

The objective of this study was to determine antibiotic residue level in beef and assess the awareness of the community and other stakeholders about beef antimicrobial residues in Nekemte town. It looked at laying the groundwork for future chances to find out more about national antimicrobial stewardship programmes and research. The data is helpful in developing a strategic rule aimed at reducing the amount of antibiotic residue found in cattle meat. The results of this investigation advance our understanding of antibiotic residue in food derived from animals. The Ministry of Health, the food industry and other organisations that wish to create regulatory programmes for Ethiopia's food industry will find the knowledge beneficial.

## 2 MATERIALS AND METHODS

### 2.1 Study area and animals

This study was conducted in Nekemte, East Wollega zone of Oromia regional state from October 2021 to November 2022. Nekemte is located at 328 km West of Addis Ababa and is situated at latitude of 9°4′ 9571 N and longitude of 36°32′9281 E and at an altitude of 2124 m above sea level. The mean annual rainfall and average temperature range from 1800 to 2200 mm and 20 to 25°C, respectively. The area receives bimodal rainfalls that were long rainy season and short rainy season. The long rainy season occurs during the months of June to September, whereas the short rainy season observed during the months of March, April and May.

### 2.2 Study populations

The animals were usually brought to Nekemte from Uke, Getema, Diga, Arjo and Sasiga markets and then purchased by restaurant owners and taken to Nekemte town municipal abattoir for slaughter. The animals are slaughtered after ante mortem examination within 1–6 h of their arrival to the slaughterhouse.

### 2.3 Study design

A cross‐sectional study was under taken in Nekemte slaughterhouses from October 2021 to November 2022. The cattle come from the surrounding district markets. In this slaughterhouse, an average of 30 heads of cattle are slaughtered daily, three times a week. The study population consisted of apparently healthy slaughtered cattle at slaughterhouses.

On each sampling day 2–3 animals were randomly selected. Kidney and muscle samples were collected from each animal in separate sterile sample containers.

### 2.4 Sample size determination

A total of 60 raw beef samples were randomly collected from Nekemte municipal abattoir found in Nekemte town. A purposive sampling was used to select this slaughterhouse, then random sampling was used to pick the beef samples from slaughterhouse, 30 kidney and 30 muscle samples from slaughterhouse.

### 2.5 Sampling method

On each sampling day, 2–3 randomly selected cattle were selected, and their muscle and kidney were used for further processing. A total of 60 samples 30 each from muscle and kidney) were taken randomly from a slaughterhouse immediately after slaughtering. Samples were packed in polyethylene bags, sealed, labelled and kept in dry ice box stored in −20°C.

### 2.6 Knowledge, attitude and practice

A total of 120 participants were included in the survey. Purposive sampling technique was used to gather information using pretested questionnaires. The purpose of the questionnaire was to collect information about the knowledge, attitudes and practices (KAP) of veterinarians, meat consumers, butcher men and the general community towards antibiotic residue in cattle. Data collection included individual interviews using questionnaires targeting 100 respondents living in Nekemte. The number of respondents was determined using the following formula: *n* = 0.25/SE^2^; where *n* is the sample size and SE is the standard error (Arsham, 2002). Considering the non‐response rate, 20% of the sample size was added to give a total of 120 participants. The non‐response rate was determined using the formula: *n*/1 − *f*, (where *f* is non‐response rate = 20%). The information collected included demographic characteristics (age, education, gender and religion), source of meat, meat preparation and consumption, knowledge on antimicrobial residues, effects of antimicrobial residues on human health, common antimicrobials and methods used to prevent antimicrobial residues.

### 2.7 Determination of antibiotic residue levels

The antibiotic residue level was performed by using high performance liquid chromatography coupled with triple‐quadrupole mass spectrometer (LC–MS/MS). In this particular study, HPLC of an Agilent 1290 Infinity II system (Agilent Technologies Ltd.) interfaced to an Agilent 6470 LC/TQ/ triple‐quadruple mass spectrometer (Freitas & Ramos, 2014). The HPLC–MS/MS equipped with an Agilent jet stream electrospray ionization source, which was operated in positive mode (AJS‐ESI+) and controlled by Mass Hunter software (Doyuk & Dost, 2023).

The antibiotics separation was chromatographically using on Phenomenex Synergi hydro‐RP, (4.6 mm × 150 mm; 4 µm, 80 Å dimensions) column with a guard cartridge system (4 × 3.0 mm^2^). The mobile phase was a binary gradient mobile phase with a flow rate set at 1.0 mL/min for a total run time of 17 min (Table 1). Methanol with 0.1% FA (Mobile phase‐A) and acetonitrile with 0.1% (v/v) (Mobile phase‐B) were used.

**TABLE 1:** Mobile phase gradient profile.

S. no.	Time in minutes	Mobile phase A (%)	Mobile phase B (%)
1.	0.00	90	10
2.	4.50	90	10
3.	4.60	80	20
4.	10.50	80	20
5.	12.00	20	80
6.	15.00	20	80
7.	17.00	90	10

### 2.8 Sample pretreatment

About 500 g of beef muscle tissue (Codex Alimentarius Commission [CAC], 2011) samples and in parallel kidney, samples of the same animals slaughtered at Nekemte town were sampled. Each sample was collected using a sterile sample collection polyethylene/plastic bag individually identified and properly labelled using labelling tape. Specific numbers, dates, and butcher's shops for the sample collection were recorded. Individually collected and packed samples were placed in an icebox during shipping to animal products and inputs testing centre of Ethiopian Agriculture Authority, and the cold chain was maintained after arrival at the laboratory center.

After removing fats, the samples were minced and homogenized using meat blender. From each homogenized sample, 4.0 g was accurately weighed, in duplicates, in a 50 mL falcon tubes and kept frozen (≤20°C) until the time of samples extraction and clean up. Before analysis, samples were allowed to defrost at room temperature.

### 2.9 Sample clean up by solid phase extraction

To each sample, 10 millilitres of the extraction solution (2 mL Na_2_EDTA‐McIlvaine buffer and 8 mL of acidified graphite carbon nitride with 0.1% FA) were added and mixed by vortexing and shaking using a calibrated solvent dispenser in sequence to the falcon tubes. Then the tubes closed tightly and mixed briefly by vortex for 30 s. Subsequently, the sample mixtures were shaken vigorously for 15 min using a wrist‐action mechanical shaker. After shaking, the 50 mL sample tubes were centrifuged for 15 min at 4500 rpm at 4°C.

Then samples purified by solid phase extraction (SPE) technique using 12 ports SPE vacuum manifold. After carefully mounting the Oasis hydrophilic lipophilic bond (HLB) SPE cartridges on the vacuum manifold, the supernatant was loaded from the 50 mL falcon tube via Oasis HLB cartridges following the proper conditioning, washing, equilibration and eluting steps.

Then the eluted solutions were directly collected into 15 mL scaled conical plastic centrifuged tubes, which were preplaced in the manifold. About 5 mL of the clean extracts were collected into another sample tubes and evaporated at 40°C under a gentle stream of nitrogen gas nearly to dryness (0.10 mL) using MultiVap 54 Lab Tech a nitrogen gas streamed sample concentrator with a 2/3 filled water bath. The sample concentrator was coupled with an online nitrogen gas generator.

Afterwards, the concentrated residues were reconstituted with 1 mL initial mobile phase, recapped and vortexed for 30 s, and centrifuged for 15′ at 4500 rpm at 4°C. Finally, clear supernatant, supposed to contain antimicrobial residues of interest, was transferred into autosampler vials and closed tightly to make them ready for injection. Finally, a 10 µL injection volume was injected in to the HPLC–MS/MS system.

A given sample was regarded as positive for tetracycline if its retention time and peak corresponded to the standard. Retention time was considered a reasonably unique identifying characteristic of a given analyte.

#### 2.9.1 LC–MS/MS method of analysis (detection and determination methods)

The analysis was performed by UHPLC of an Agilent 1290 Infinity II system (Agilent Technologies Ltd.) interfaced to an Agilent 6470 LC/TQ/ triple‐quadrupole mass spectrometer (MS/MS) equipped with an Agilent jet stream electrospray ionization source, which was operated in positive mode (AJS‐ESI+) and controlled by MassHunter software. Antimicrobials separation was chromatographically achieved on Phenomenex Synergi hydro‐RP (4.6 mm × 150 mm; 4 µm, 80 Å dimensions) column with a guard cartridge system (4 × 3.0 mm^2^). The mobile phase was a binary gradient mobile phase with a flow rate, which was set at 1.0 mL/min for a total run time of 17 min (Table 1). Methanol with 0.1% FA (mobile phase‐A) and acetonitrile with 0.1% (v/v) (mobile phase‐B) were used.

### 2.10 Data analysis

The obtained data was captured in Excel and imported into SPSS version 20 software, descriptive and inferential statistics (*t* test) were used to analysis data. Categorical variables were expressed by proportions and their significance was assessed, when appropriate using chi‐square (*χ* ^2^) test, and a *p*‐value of ≤0.05 was considered statistically significant. Continuous variables were expressed by means and standard deviations and assessed for statistical significance using the Kruskal–Wallis chi‐square test.

## 3 RESULTS

The KAP of the cattle handlers regarding antibiotics residue in dairy and beef product was evaluated.

A majority of respondents (70.8%) were in the 20–40 age range, whereas 29.2% were older than 41 years. Male respondents made up 66.7% of the sample, whereas female respondents made up 33.3%. Protestants made up (57.5%) of the respondents followed by orthodox (31.7%) and Muslims (10.8%). In terms of respondents’ knowledge of antimicrobial residue, there is a significant difference between those university a degree and those with lower education level *p* < 0.05 (*p* = 0.00), but there is no significant difference in respondents’ knowledge based on age or sex (*p* = 0.88) (Table 2).

**TABLE 2:** Demographic characteristics of respondents (*n* = 120).

Demographic parameters studied	Category	Frequency (*n*)	Percentage
Age	20–40	85	70.83
	>41	35	29.17
Gender	Male	80	66.67
	Female	40	33.33
Education level	No formal education	17	14.17
	Primary	25	20.83
	Secondary	27	22.50
	Diploma	17	14.17
	Degree	29	24.17
	Masters	5	4.16
Job	Cattle handler	36	30.00
	Butcher	24	20.00
	Farmer	60	50.00
Religion	Orthodox	38	31.67
	Protestant	69	57.50
	Muslim	13	10.83

### 3.1 Respondent's knowledge on antimicrobial use and drug residues

Most of the respondents (52.50%) know drug residues. About 54.17% of the respondents knew the effects of residues in human health. The majority (56.67%) were aware of that economic impact of antibiotic residues. About 74% of the respondents know about drug withdrawal period, whereas 25.8% do not know about withdrawal period and its health effects. Only 40% of respondents know methods to control and prevent drug residues in beef meat, whereas 60% do not. Knowledge on antimicrobial drugs increased as the education level increased. The differences were strongly significant *p* < 0.05 (Table 3).

**TABLE 3:** Respondents’ knowledge on antimicrobial use and drug residues (*n* = 120).

Knowledge level assessed	Response	Frequency	Percentage
Do you have any awareness on drug residues	Yes	63	52.50
	No	57	47.50
Do you use antibiotics for treatment	Yes	85	70.8
	No	35	29.2
Do you use antibiotics for prevention	Yes	85	70.8
	No	35	29.2
For what purpose do you use antibiotic use	Fattening only	15	12.50
	Treatment of disease only	33	27.50
	Prevention of disease only	10	8.30
	Both fattening and disease treatment	25	20.83
	Fattening, disease treatment and prevention	47	39.17
Have you heard drug residue	Yes	51	42.50
	No	69	57.50
Have you heard about withdrawal period	Yes	31	25.83
	No	89	74.19
Do you think antimicrobial residue have effects health	Yes	65	54.17
	No	55	45.83
Do you think antimicrobial residue have effects on the economy of the country	Yes	68	56.67
	No	52	43.33
Do you think we can control the effects of antimicrobial residue on health	Yes	48	40.00
	No	72	60.00

### 3.2 Respondent's attitudes on antimicrobial use and drug residues

Approximately 58.33% of participants disagreed that antibiotics used to treat animals end up in animal tissue. About 52.5% of respondents agreed that eating meat contaminated with antibiotics has an adverse effect on one's health. The majority of respondents (68.33%) concurred that a veterinarian's post‐treatment guidance may lower the likelihood of antibiotic residue. The majority of respondents (60.83%) thought that heating meat could lower the possibility of antibiotic residue (Table 4).

**TABLE 4:** Attitudes of the respondent on the antibiotic residue in meat (*n* = 120).

Attitudes towards antimicrobial residue	Response	Frequency	Percentage
Do you agree that antibiotic residue present in animal tissue	Agree	47	39.67
	Not agree	73	60.33
Do you agree that antibiotic residue in animal source food have public health effect	Agree	63	52.50
	Not agree	57	47.50
Do you think that post‐treatment advice is essential in preventing the heath effect of antibiotic residue	Agree	81	67.50
	Not agree	39	32.50
Do you agree that cooking meat reducing risk the risk of antibiotic residue on health	Agree	73	60.83
	Not agree	47	39.17
Do you agree that the health risk of antibiotic residue is preventable	Agree	62	51.67
	Not agree	58	48.33

### 3.3 Respondents’ practice about beef

Most respondents purchase meat from butcheries (80%), followed by back yard slaughter (16.67%). Most of the respondents (72.50%) purchased meat, whereas others purchase offal's such as liver (15.83%) and kidney (7.50%). Respondents were reply for consuming meat one to two times only per month where cooking was the most common method (86.67%) of preparation (Table 5).

**TABLE 5:** Respondents’ practice about beef (*n* = 120).

Practice level assessed	Category	Frequency	Percentage
What are your source of meat for consumption	Supermarket	4	3.33
	Butcheries	96	80.08
	Local	20	16.67
Frequency of meat consumption per month	Once	52	43.33
	Twice	45	37.50
	Three times	14	11.67
	≥4 times	9	7.50
Which part of meat you frequently consume	Muscle	87	72.50
	Liver	19	15.83
	Kidney	9	7.50
	All part	5	4.17
How do you eat the meat	Cooking	104	87.67
	Eating raw	14	11.67
	By freezing	2	1.66
Do you always use e similar drug	Yes	68	56.67
	No	52	43.33

### 3.4 Detection and quantitative analysis by HPLC–MS/MS

**FIGURE 1:**
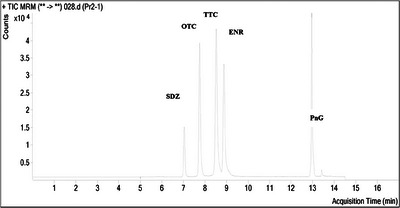
Chromatograms (the visual output of the chromatograph) of tetracycline. *Note*: Sulfadiazine (SDZ), oxytetracycline (OTC), tetracycline (TTC), doxycycline (DXY) and penicillin G (PnG) in meat matrices.

Samples collected from beef cattle were analysed by HPLC for quantification of antimicrobial residue. Retention time was considered a reasonably unique identifying characteristic of a given analyte. Figure 1 shows chromatograms (the visual output of the chromatograph) in which *x*‐axis is the retention time and the *y*‐axis is a signal obtained by ultra‐violate diode array detector corresponding to the amount of antibiotics existing in the system. The peaks are characteristic of their identity, with a distribution around the mean position (apex of the peak) that is characteristic of the kinetic properties of the chromatographic system. The area inscribed by the peak is proportional to the amount of substance separated in the chromatographic system. To get the concentration of an antibiotic sample, a reference standard of a known concentration was injected into the HPLC, and the concentration of the sample was extrapolated from the curve peak area.

The *x*‐axis is the retention time and the *y*‐axis is a signal obtained by UV diode array detector corresponding to the amount of antibiotics existing in the system. The peaks are characteristic of their identity, with a distribution around the mean position (apex of the peak) that is characteristic of the kinetic properties of the chromatographic system. The area inscribed by the peak is proportional to the amount of substance separated in the chromatographic system. To get the concentration of an antibiotic sample, a reference standard of a known concentration was injected into the HPLC, and the concentration of the sample was extrapolated from the curve peak area (Table 6).

**TABLE 6:** The residue analysis values in beef meat.

Residue tested for	RV (%)	LOD (µg/kg)	LOQ (µg/kg)	MRL (µg/kg)
Sulfadiazine	101.2	1.79	5.95	100
Oxytetracycline	100.3	2.33	7.76	100
Tetracycline	106.6	1.99	6.62	100
Doxycycline	95.9	2.39	7.95	100
Enrofloxacin	105.7	1.99	6.63	100
Penicillin G	95.9	2.53	8.44	50

Abbreviations: LOD, limit of detection; LOQ, limit of quantification; MRL, maximum residue limit; RV, recovery value.

### 3.5 Proportion of tetracycline antibiotic residues

Out of the total 60 meat samples, 15 (50%) and 60 (100%) had detectable oxytetracycline and tetracycline residues, respectively, whereas doxycycline, sulphadiazine, penicillin G and enrofloxacin were not detected. The ranges for tetracycline residue levels from individual organs were 0.12 to 463.35 µg/Kg for kidney and 0.41 to 354.55 µg/Kg for muscle (Table 7).

**TABLE 7:** Occurrence of antibiotic residues in kidney and muscles (*n* = 30).

	Antimicrobial residues prevalence (Test +ve)						
Sampled organ	SDZ (%)	OTC (%)	TTC (%)	ENR (%)	DXY (%)	PnG (%)	Prevalence (%)
Kidney	0 (0.0)	9 (30)	30 (100)	0 (0.0)	0 (0.0)	0 (0.0)	39 (130)
Beef	0 (0.0)	6 (20)	30 (100)	0 (0.0)	0 (0.0)	0 (0.0)	36 (120)
Total	0 (0.0)	15 ((50)	60 (100)	0 (0.0)	0 (0.0)	0 (0.0)	75 (125)

Abbreviations: DXY, doxycycline; OTC, oxytetracycline; PnG, penicillin G; SDZ, sulfadiazine; TTC, tetracycline.

## 4 DISCUSSIONS

Veterinary medications are currently widely utilized to treat and prevent illnesses in animals, encourage healthy growth in animals, and increase feed conversion rates. Nevertheless, veterinary medication use in animals raised for food production may leave parent chemical and/or metabolite residues in food items that are toxic to humans (Getahun et al., 2023). The issue of antibiotic residues in meat is a serious problem that is not effectively addressed in low and middle income countries including Ethiopia. The safety of foods of animal sources regarding drug residues receives suboptimal attention in the country and concerns on the public health impact such as antibiotic resistance of bacteria strains in humans and animals are growing. Antibiotic overuse and unprocessed antibiotic disposal are putting the environment and its people in danger of a major health hazard. The environment is with an abundance of bacteria and its genes that are resistant to many antibiotics; thus, the effective removal of antibiotics must be addressed right away (Apreja et al., 2022).

This study revealed that knowledge and practice regarding antibiotic residues were generally moderate among cattle handlers in the study area. A large proportion of the participants understood that the presence of antibiotics in meat was a result of antibiotic residues in animal meat, and this agrees with the report by Mgonja et al. (2018), which showed 35% of the respondents were aware about antibiotic residue in meat. Two thirds of the participants confirmed waiting a while after administering antibiotics before slaughtering, and more than half of them had limited knowledge of the concept of withdrawal period. This is consistent with Babapour et al. (2012) report, which found that non‐adherence to drug withdrawal requirements was linked to certain risk factors for food contamination. The poor knowledge observed in this study suggests that collaborative efforts in improving sensitization or educational programmes on antimicrobial stewardship are crucial.

Oxytetracycline was indiscriminately used in the study areas. About half of the cattle handlers reported that they are using antibiotics without veterinarian's prescription. In the same vein, about half of the participants indicated self‐prescribing and repeating antibiotic treatment previously administered by veterinarians for animals when presenting similar signs. This is in line with the study conducted by Alhaji and Ishola (2018) among pastoralists in North‐Central Nigeria who practiced self‐prescription and administration without professionals’ consultations. Farmers or cattle handlers result to self‐medication due to lack of adequate and high cost of veterinary services as reported by Olasoju et al. (2021). This widespread and unrestricted usage of different antibiotics in food animals without adequate diagnosis, prescription and supervision of veterinarians has contributed greatly to the deposition of residues in animal products (Olatoye & Ehinmowo, 2011). Kinsella et al. (2009) reported that the use of antibiotics in animal agriculture is significantly higher than in human medicine, which could result in antibiotic residue and hence antimicrobial resistance. Study conducted by Muriuki et al. (2001) in Nairobi and surrounding areas reported 45.6% detectable levels for tetracycline.

Overall, these findings suggest that approximately about 3.33% and 10% of muscle and kidney samples collected from Nekemte town had oxytetracycline residues above maximum residual limits, which could have potential health implications for consumers. The high prevalence of oxytetracycline residues observed in the current study probably reflects cattle have sold for slaughter whilst under a therapeutic or prophylactic regimen or animals being slaughtered before the end of the withdrawal period (Aiello & Moses, 2010). Slightly lower report exists elsewhere as compared to the current finding. Duong 2005 reported lower residue level of tetracycline (5.52%) from pork samples. Moreover, Patyra et al. (2020) reported higher residue level 63%–93% from samples of liquid manures and spiked pig and poultry excrement.

The prevention levels of antibiotics beyond maximum residue limits require combined and coordinated effort among government agencies, veterinarians and livestock producers. As it obtains in high income countries, the avoidance of meat residues in the livestock industry should take an on‐farm team effort that begins with the veterinary–client–patient‐relationship (The College of Veterinarians of Ontario [CVO], 2020). The cattle farm owner/manager/herdsman must work with the farm veterinarian to develop treatment protocols that address the judicious use of antibiotics. Once a decision is made to use antibiotics, protocols must then be put in place to guide employees on the safe way to handle this animal to prevent inadvertent meat residues from occurring. Essentially, treated animals should be identified and antibiotic use must be recorded to prevent residues (CVO, 2020).

In this study, participants’ demography such as age (*p *< 0.05) and gender (*p *< 0.05) were significantly associated with knowledge on antibiotic residues. Education was expected to have a positive association with knowledge as reported by Eltayb et al. (2012), Pham‐Duc et al. (2019) and Ibrahim et al. (2020). The poor knowledge observed among the young educated cattle stake holders could have resulted from lack of awareness of the issues of antimicrobial residues, carefree attitude as well as lack of experience compared to the older stake holders who might have gained experience over the years. In addition, female respondents were likely to have poor knowledge of antibiotic residues than their male counterparts and this is not in agreement with the reports of Olasoju et al. (2021) in which female participants were more likely to have better knowledge than their male counterparts. On the contrary, a previous report by Ibrahim et al. (2020) documented male participants were more likely to have better knowledge than female counterparts.

In the current study, 50% of oxytetracycline and 100% of tetracycline residue were detected. This finding is lower to report by Bedada and Zewde (2012) at Ethiopia who reported 71.3% oxytetracycline residue level. The current study revealed higher value to the report from Iran who indicated 75% residue level (Baghani et al., 2019). Furthermore, it may indicate that the recommended withdrawal time may not have been respected before slaughtering of the animals. In this study, doxycycline, enrofloxacin and penicillin G were not detected in all samples, which are consistent with the findings by Bedada and Zewde (2012).

Furthermore, the current study found much lower concentration of oxytetracycline in the kidney than another finding reported in Addis Ababa, Adama and Bishoftu slaughterhouses, which reported 99.02, 109.35 and 112.53 µg/respectively (Uma & Ashenef, 2023). The variation in the levels of oxytetracycline residues in the tissue samples could be due to exposure of the animals to antibiotics weeks or even days before slaughter, unauthorized use of the antimicrobials, over use of the drug, inadequate knowledge of the farmers and/or failure to apply instructions on the drug label. In addition, this may be due to the unreasonable use of large quantities of drugs without a professional prescription, the relatively cheap intake of antibiotics, and the inappropriate intake of antibiotics. Again, this antibiotic is widely used in these areas of research, probably due to their affordability, accessibility and broad‐spectrum effect. In addition, this may be due to lack of awareness and outreach, which may lead to drug abuse and overuse and may result in failure to observe discontinuation periods. This indicates that there is a need to take appropriate action by the concerned bodies to protect the consumer's health. The current study also recommends further research, particularly to assess the factors that increase the risk of antibiotic residues.

### 4.1 Limitation of the study

The cost of analysis is somewhat expensive, and some butcher houses are not willing to give samples for analysis of antibiotic residue. Additionally, only a few antibiotic analysis protocols were found in the country.

## 5 CONCLUSIONS AND RECOMMENDATIONS

The present survey contributes to the better understanding of the current status of cattle handlers’ levels of knowledge and practice regarding antibiotic residues in current study area. The use of antibiotics to bring about improved performance in growth and feed efficiency, to synchronize or control of reproductive cycle, and to improve breeding performance also often leads to harmful residual effects. It may be due to inadequate awareness and insufficient extension activities that can lead to misuse and overuse of the drug, and possibly failure to observe withdrawal periods that necessitate immediate action. The study indicated the presence of high antimicrobial residue and hence exposes for antimicrobial resistance of pathogens warranting coordinated effort to mitigate its health effect on the animal and hence human being.

## AUTHOR CONTRIBUTIONS

Mathewos Tafesse and Debela Abdeta proposed the idea, write the proposal, methodology, data analysis and write original draft. Balachew Bacha supervised and validated all procedures during data collection and accomplishes final correction to the work. Debela Abdeta also writes final draft and takes responsibility in manuscript submission process.

## CONFLICT OF INTEREST STATEMENT

The authors declare there are no conflicts of interest.

## FUNDING INFORMATION

None.

### ETHICS STATEMENT

Ethical approval was obtained from the Research Ethics Committee of the School of Veterinary Medicine, Wallaga University dated 13 November 2021 with minute number SVM.RERC/0029 before starting data collection. Written informed consent was obtained from cattle handlers, butcher men and meat users of above 18 years prior to the commencement of the study. The Research Ethics Committee of the School of Veterinary Medicine, Wallaga University approved all aspects of this study, including the informed oral consent process. Communicative letter was written to Nekemte municipality explaining the objective of this study. Data was collected after explaining the aims and methodology of the study. It is assured that information collected was anonymous and that it was only for research purpose.

### PEER REVIEW

The peer review history for this article is available at https://publons.com/publon/10.1002/vms3.1459.

## Data Availability

The data that supports this finding is with the corresponding author and can be provided up on request.
